# Formulation and evaluation of Piroxicam nanosponge for improved internal solubility and analgesic activity

**DOI:** 10.1080/10717544.2023.2174208

**Published:** 2023-02-06

**Authors:** Dalia A. Gaber, Mahasen A. Radwan, Danah A. Alzughaibi, Jenan A. Alail, Rafa S. Aljumah, Reema M. Aloqla, Sara A. Alkhalifah, Siham A. Abdoun

**Affiliations:** a Department of Pharmaceutics, College of Pharmacy, Qassim University, Buraidah, Kingdom of Saudi Arabia; b Pharmacy Practice, Clinical Pharmacy Department, Faculty of Pharmacy, Egyptian Russian University, Badr City, Cairo, Egypt; c College of Pharmacy, Qassim University, Buraidah, Kingdom of Saudi Arabia

**Keywords:** Nanosponges, crosslinker, nanocarrier, cyclodextrin, analgesics, drug efficacy, polymer

## Abstract

Cyclodextrin nanosponges are solid nanoparticles, designed by cross-linking of cyclodextrin polymer; it has been used widely as a good delivery system for water insoluble drugs. The aim of this study is to enhance the solubility of Piroxicam (PXM) using β-Cyclodextrin based nanosponges formulations. PXM nanosponge (PXM-NS) formulations were prepared using β-cyclodextrin and carbonyldiimidazole as a cross linker, three ratios of β-cyclodextrin to crosslinker in addition to three drug to nanosponges ratios were tested. Piroxicam nanosponge formulations were characterized for its particle size, zeta potential, physical compatibility and in vitro release. Stability studies at three temperatures (4 °C, 25 °C and 40 °C) were done for optimal formula. Finally, the in vivo analgesic activity and pharmacokinetic parameters of the optimal formula were conducted. The optimized PXM-NS formula (PXM-NS10) showed particle size (362 ± 14.06 nm), polydispersity index (0.0518), zeta potential (17 ± 1.05 mV), and %EE (79.13 ± 4.33). The dissolution study showed a significant increase in the amount of PXM dissolved compared with the unformulated drug. Stability studies confirmed that nanosponge showed accepted stability for 90 days at 4 °C and 25 °C. In vivo analgesic studies verified that there was a significant enhancement in the analgesic response to PXM in mice, and 1.42 fold enhancement in the relative bioavailability of PXM-NS10 as compared to commercial tablets. Nanosponge prepared under optimal conditions is an encouraging formula for increasing the solubility and therefore the bioavailability of Piroxicam.

## 1. Introduction

**Figure 1.**
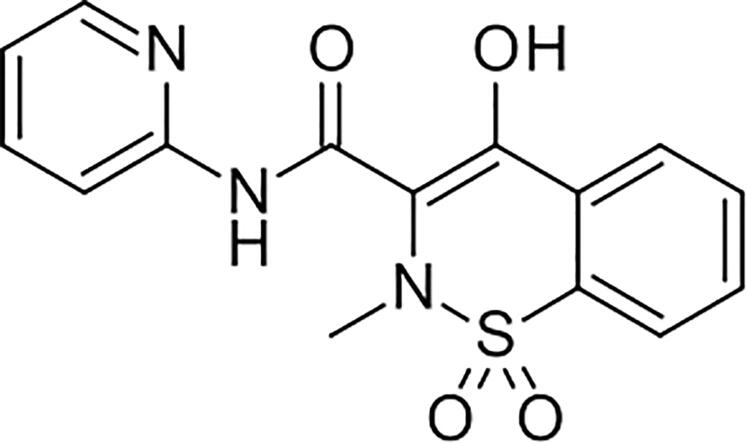
Chemical structure of Piroxicam.

Oral route for deliver drugs is the favored route compared to other routes. Unlike the other routes of medication, it permits ease of administration moreover it is the natural and the highly suitable way for substances to be delivered into the human body (Amidon et al., 1995; Merisko-Liversidge et al., 2003; Gaber et al., 2022a). In spite of these privileges of oral route many drugs having difficulties such as low solubility, narrow absorption window and poor bioavailability (Serajuddin, 2007). About seventy percent of the drugs used in the pharmaceutical formulations are either slight or insoluble in water (Merisko-Liversidge et al., 2003). To overcome these restrictions different drug delivery systems such as nanoemulsions, solid dispersions, microemulsions, nanosuspensions, nanosponges etc. have been designed to increase the solubility and improve the bioavailability (Kawabata et al., 2011). Nanosponges can be described as a hyper cross linked structure composed of a cyclodextrin (CD) in three dimensional networks; they are formed by reacting CD with a suitable cross-linking agent such as diphenyl carbonate or carbonyldiimidazole (Ansari et al., 2011). Cyclodextrins (CDs) are composed of cyclic oligosaccharides used widely in pharmaceutical industry during the last 20 years (Attia et al., 2007). It has a great ability to form noncovalent soluble complexes, also they are suitable as efficient excipients for enhancing the solubility, delivery, and bioavailability of drugs in many new dosage forms (Bencini et al., 2008; Bertino Ghera et al., 2009). Cyclodextrin polymer has a bucket shape with a hydrophobic central cavity and a hydrophilic outer surface. Based on its structure CDs have the ability to host hydrophobic drugs Its cavities have various width varied between 0.3 and 0.7 nm which helps the formation of complexes with the hosted drug molecules and hence improve its solubility (Pushpalatha et al., 2018). Several types are present from CD include, α-CD, β-CD, and γ-CD which composed of 6, 7, and 8 units of d-glucopyranose respectively, with α −1,4 linkages (Khalid et al., 2011). The low cost, the simple synthesis, and the suitable cavity size for the inclusion of drug molecules with different size help to a wide use of β-cyclodextrin in pharmaceutical applications (Mognetti et al., 2012). Drugs having problems such as low water solubility and/or low stability will show a reduction in its therapeutic activity (Suresh et al., 2007). In order to overcome these problems, β-CD used to form an inclusion complex with these drugs. Hence, it enhances the bioavailability of poorly soluble drugs by increasing their solubility and therefore its bioavailability (Shikov et al., 2009). Piroxicam (PXM) is one of the most efficient nonsteroidal anti-inflammatory agents. PXM has a potent analgesic and anti-inflammatory activity. It is widely used for the treatment of osteoarthritis, rheumatoid arthritis, and acute pain, inflammation of joints, and for pre and post-surgery pain (Figure 1). PXM belongs to Class II drugs which have a low solubility and high permeability (Wu et al., 2009). Because of its low solubility; it has poor bioavailability after oral use (Prabhu et al., 2005). The formation of inclusion complexes with β-CD is useful method to overcome solubility problems. Many studies have reported the ability of nanosponge dosage form to improve the solubility and hence the oral bioavailability of other poorly soluble drugs such as itraconazole, dexamethasone, tamoxifen and resveratrol (Swaminathan et al., 2007; Khalid et al., 2011; Swaminathan et al., 2013; Torne et al., 2013). Thus, the aim of this study was to improve the oral solubility of piroxicam through incorporation into β-CD nanosponges. Twelve formulations were designed based on the use of four β-CD/cross linker ratios (1:2, 1:4, 1:6, and 1:8) and three drug/β-CD nanosponges ratios (1:2, 1:4, and 1:8). The physico-chemical characterizations of the PXM-NS formulations along with their in vitro release pattern are reported. Finally, the analgesic activity and the pharmacokinetic profile of the optimal formula were studied (Adams et al., 1969).

## 2. Material and method

### 2.1. Materials

Piroxicam (PXM) was a free sample gift from El-Nile Company for Pharmaceutical & Chemical Industries, Egypt. Brexin® 20 mg tablets, Chiesi pharmaceutical company, Italy. β-Cyclodextrin (β-CD) Mwt 1134.98 was purchased from ‘Shangdong Binzhou Zhiyuan Biotech’ Co., Ltd., China. Carbonyldiimidazole was obtained from Sigma-Aldrich, Darmstadt, Germany. Dimethyl formamide (DMF) from Merck, Mumbai, India. Methanol, Ethanol, and Acetonitrile were HPLC grade. Other chemicals were of analytical grade and were used as obtained.

## 3. Method

### 3.1. Synthesis of β-Cyclodextrin nanosponges

β-Cyclodextrin based nanosponges were designed using carbonyldiimidazole as cross linker in four ratios (i.e. 1:2, 1:4, 1:6, and 1:8). Briefly, β-CD was dissolved in 100 ml of DMF. Then carbonyldiimidazole was added and the reaction was kept for 6 hours at 100 °C. The formed cross linked β-Cyclodextrin was allowed to cool, filtered and washed with an excess amount of deionized water to remove any unreacted DMF. The residue was then dried in an oven at 60 °C for 6 hours and subsequently grounded in a mortar. The powder was then extracted with ethanol using Soxhlet method to remove unreacted reagents. The formed β-CDNS were lyophilized and kept at desiccator 25 °C until further use (Dubes et al., 2003; Adams et al., 1969).

### 3.2. Preparation of PXM loaded β–CD nanosponges

PXM loaded nanosponges were prepared at three different weight ratios; 1:2, 1:4 and 1:8 (Drug: β–CD nanosponges w/w). β–CD nanosponges and PXM powder were mixed in the different ratios as shown in Table 1 and the mixture was suspended in 100 ml deionized water. Then, the mixture was sonicated for 10 min and then stirred at 75 rpm for 24 h. The aqueous suspension was centrifuged (Lab Benchtop MR202, India) for 10 min at 2000 rpm to separate the unreacted drug. The supernatant was then lyophilized. The PXM-β-CD NS formulations were pulverized and stored in a desiccator at room temperature until further use (Dubes et al., 2003).

**Table 1.** PXM-β–CD NS formulation, particle size, polydispersity index, zeta potential and % entrapment efficiency (% EE).

Formula code	β-CD: Cross linker ratio	PXM: β-CD ratio	Particle size ± SD (nm)	PI	**ZP** **(-mV)**	EE (%)
PXM-NS1	1:2	1:2	322 ± 10.26	0.0548	17 ± 2.34	35.33 ± 3.12
PXM-NS2	1:2	1:4	328 ± 16.05	0.0581	20 ± 1.21	37.24 ± 1.25
PXM-NS3	1:2	1:8	330 ± 14.04	0.0353	15 ± 3.31	36.63 ± 5.27
PXM-NS4	1:4	1:2	347 ± 15.12	0.0433	15 ± 2.12	50.93 ± 3.02
PXM-NS5	1:4	1:4	351 ± 13.02	0.0421	12 ± 3.10	49.80 ± 6.01
PXM-NS6	1:4	1:8	349 ± 17.16	0.0433	10 ± 1.11	52.33 ± 5.22
PXM-NS7	1:6	1:2	351 ± 20.16	0.0452	13 ± 1.23	52.33 ± 5.22
PXM-NS8	1:6	1:4	345 ± 18.20	0.0361	15 ± 2.60	57.52 ± 4.53
PXM-NS9	1:6	1:8	355 ± 15.12	0.0351	14 ± 2.00	65.2 ± 4.14
PXM-NS10	1:8	1:2	362 ± 14.06	0.0518	17 ± 1.05	79.13 ± 4.33
PXM-NS11	1:8	1:4	359 ± 18.10	0.0398	10 ± 3.22	71.90 ± 1.22
PXM-NS12	1:8	1:8	369 ± 14.01	0.0423	15 ± 2.31	72.81 ± 3.12

### 3.3. Characterization of PXM-β–CD nanosponges

#### 3.3.1. Particle size, polydispersity, and zeta potential determination

The mean particle size, polydispersity index (PI) and zeta potential of designed PXM nanosponge formulations were determined using zetasizer (DTS Ver. 4.11, Malvern Instruments, UK). All PXM-NS formulations were suspended in deionized water for assessment (Gaber et al., 2022b).

#### 3.3.2. Determination of % encapsulation efficiency

Weighs of PXM-NS formulations were crushed, dissolved in 20 mL methanol, stirred for 60 minutes, the supernatant was withdrawn, diluted and analyzed spectrophotometrically at a wavelength of 335 nm (Jenway, UK). The encapsulation efficiency was calculated from the following equation:

$$\text{\% EE =}\frac{\text{The actual amount of drug}}{\text{The theoretical amount of the drug }}*100$$

The test was done in triplicate and the results were expressed as mean ± SD (Trotta et al., 2012).

#### 3.3.3. Saturation solubility study

Saturation solubility study of PXM in the PXM-NS formulation was done. Briefly, 10 mg of PXM-NS formulations was suspended in 1 ml distilled water and packed into 2 mL centrifugation tubes and centrifuged at 15000 rpm for 1 h. The supernatant was filtered through Whatman filter paper (45 µ) and PXM content was determined by spectrophotometer at λ_max_ 335. The test was conducted in comparison with pure drug, the saturation solubility of an equivalent amount of PXM in 1 mL distilled water was determined to estimate the impact of CD-NS entrapment on PXM solubility (Haroon et al., 2017).

#### 3.3.4. Study of nanosponge morphology by scanning electron microscope method (SEM)

The surface morphology of the selected formula of PXM-NS was examined by SEM ‘Metler Toledo, Tokyo, Japan’. All samples were fixed in an individual stub and coated uniformly with gold and photographed.

#### 3.3.5. Fourier transform infrared–FTIR-spectroscopic analysis

For identifying the probable interaction between PXM and CD in nanosponge formulations, FTIR studies were conducted using FTIR spectrophotometer (Shimadzu 1800, Japan). Samples were blended with Potassium bromide and pellets were made by press (Shah et al., 2016).

#### 3.3.6. Differential scanning calorimetry–DSC-analysis

Thermal behavior of PXM and selected PXM-NS formulation were considered using differential scanning calorimetry (DSC) (Mettler Toledo, Switzerland). In an aluminum pan, 5 mg samples were heated at a regular scanning rate (10 °C/min) over temperature range 40–200 °C under a nitrogen flow rate 50 mL/min (Shah et al., 2016).

#### 3.3.7. In vitro drug release studies

In vitro drug release studies of PXM from β–CD NS formulations were carried out for formulations with encapsulation efficiency 50% and more using an eight station USP dissolution apparatus II (Logan Instruments Corp, USA). The dissolution studies were carried out in 900 ml of simulated gastric fluid (pH 1.2) for 2 hours then the media was shifted to pH 7.4 for the next 10 hours. The study was carried out at 37 ± 0.5 °C and 75 rpm. Five ml samples were withdrawn from the dissolution medium at each time intervals, passed through Whatman filter paper (45 µ) and analyzed spectrophotometrically at 335 nm after suitable dilution. The experiment was carried out in triplicate and the mean values were plotted versus time. The results were expressed as percentage of the cumulative amount of drug released as a function of time (Patel and Patel, 2012).

#### 3.3.8. Kinetic study

The *in vitro* release data were analyzed according to different kinetic models; Zero-order, First-order kinetics, Higuchi diffusion and Korsmeyer-Peppas models (Bolton and Bon, 2004).

#### 3.3.9. Stability study

Stability study was conducted to determine the possible drug degradation and/or particles aggregation upon storage in different temperatures. The stability of the selected formula of PXM-NS was measured by storing samples for 90 days at three temperatures (4 °C, 25 °C, and 40 °C). At constant time intervals (0, 15, 30, 45, 60, and 90 days) drug content was determined by the method described before and the size was determined using the Zetasizer (Deng et al., 2010).

#### 3.3.10. Study of the analgesic activity of PXM-NS using hot plate method

The *in vivo* analgesic activity of the selected PXM-NS formula (PXM-NS10) was investigated by hot plate method. Three groups of Swiss-albino mice weighing 30–40 g were used each group consists of six mice. Group I received weighted amount of PXM-NS10 formula suspended in 0.5 mL distilled water, group II received equivalent amount of PXM from commercial tablet crushed and suspended in 0.5 mL distilled water, and finally group III used as a control, which received 0.5 ml saline. Food was withdrawn 6 hours before the experiment for all groups with free access to water before and during the experiment. The in vivo experiments were carried out according to the ethical guidelines established and approved by the committee of research ethics in Qassim University. The mice were placed on the hot plate analgesia meter adjusted at temperature 51 ± 1 °C, that the plate surface is hot enough to cause discomfort sensation without injured the paw tissue. Mice in group I & II were received orally the calculated amount of PXM-NS and commercial tablets 30 minutes before the beginning of the test. The time taken till the mice start to jump and/or lick fore-paw was recorded (reaction time). The mice did not left for more than 25 seconds in each trial to avoid tissue damage. The reaction time for the analgesic effect of PXM in nanosponge and commercial tablets were measured after 0.5, 1, 2, 3, 4, 5, 6, 7, and 8 hours (Kumar et al., 2019).

### 3.4. Statistical analysis

All results were stated as mean ± standard deviation (SD) for *in vitro* tests and as means ± SE for *in vivo* tests. One way analysis of variance (ANOVA) test followed by the Duncan’s multiple comparison tests were used. A probability value (p) less than 0.05 was considered to be a significant value.

#### 3.4.1. In vivo pharmacokinetic study

##### 3.4.4.1. Study design

The *in vivo* pharmacokinetic study was conducted for optimum formula (PXM-NS10) on male Wistar rats (220–250 gm). Eighteen rats were housed with free access to water for 12 h before the experiment and during the experiment. In a random and equal manner, the rats were divided into three groups. Calculated weights of the formula based on animal weight was suspended in 1 ml distilled water and given via oral gavage to the first group. Market tablet was cursed and the weight of an equivalent amount of drug was suspended in distilled water (1 ml) and given to rats in the second group. One ml of normal saline was given to rats in the third group. The blood samples (0.5 ml) were withdrawn from the tail at 0, 0.5, 1, 2, 3, 4, 6, 8, 12, and 24 hours after dosing in a heparinized tube (Kumar et al., 2019; Mani et al., 2021). The collected blood samples were centrifuged (Hettich Zentrifugen, Germany) at 4000 RPM for 10 minutes and stored in freezer at −20 °C until further work. PXM concentration in plasma samples was assayed by means of HPLC technique (Amberkar et al., 2011).

##### 3.4.4.2. Chromatographic analysis

Determination of PXM concentration in the plasma samples was conducted according to the method reported by Burcea-Dragomiroiu, G. et al analysis after verification (European Federation of Pharmaceutical Industries Association and European Centre for the Validation of Alternative Methods, 2001). Briefly, the study was performed on a Waters Acquity HPLC™ (Waters Corp., Milford, MA, USA) equipped with UV detector. Tenoxicam was used as an internal standard (IS) and the output was detected at λ_max_ 330 nm. The composition of the mobile phase was a trifluoroacetic acid and acetonitrile mixture (60:40 v/v) ratio supplied isocratically with a flow rate of 1.1 ml.min^−1^. The analysis assay was validated for selectivity, linearity, precision, accuracy, and carry over, extraction recovery and stability shortly before the study started.

### 3.5. Calculation of PXM concentration and statistical analysis

Pharmacokinetic parameters were calculated from the plasma concentrations versus time curve. Plasma concentrations of PXM are calculated as the mean ± standard error. The maximum plasma concentration (C_max_), the time to reach the maximum concentration (t_max_). The terminal elimination rate constant (K_el_) was measured by a linear regression analysis of the terminal portion of the log-linear plasma concentration/time profile of PXM. The extent of absorption (AUC_0–t_) was calculated using linear trapezoidal rules.

The relative bioavailability (F) with the commercial product was calculated using the following equation:

 F = AUC_test_/AUC_ref_ × 100(3)

All statistical differences in data were evaluated by IBM SPSS Statistics 20 (Armonk, NY, USA) using one-way ANOVA with extended LSD post hoc test for the determined pharmacokinetic parameters, and P-value < .05 was considered significant.

## 4. Result and discussion

In the study, PXM was entrapped in β-CD nanosponges using carbonyldiimidazole as a cross linker. Twelve formulations were designed based on using four different ratios of cross linker and three different ratios of drug to nano sponges (i.e. 1:2, 1:4, and 1:8). All other formulation parameters were kept unvaried during the study. Particle sizes, zeta potential, encapsulation efficiency, and saturation solubility of the designed formulations were studied. In vitro release and in vivo study for the analgesic activity and kinetic parameters of PXM were investigated for selected formulations.

### 4.1. Particle size (PZ), polydispersity index (PI), and zeta potential (ZP)

The particle size, polydispersity index, and zeta potential (ZP) of the formed PXM-NS formulations were measured for fresh samples using Zetasizer Ver. 5.11 Malven. The mean PZ, PI, and ZP of PXM-NS formulations are shown in Table 1. The Results show that the ratio between CD and cross linker plays an important role in nanosponge size. PXM-NSs particle size range was between 322 ± 10.26 and 369 ± 14.01 for PXM-NS1 and PXM-NS12, respectively. The results showed that there was a direct relationship between cross linker concentration and particle size was noticed, the smallest size was for NS formulations with the lowest cross linker concentration. A similar observation was reported by H.V. Gangadharappa et al. who mention a direct relation between the celecoxib nanosponge particle size and crosslinker concentration. Other studies stated an increase in the particle size with a decrease in crosslinker concentration which could be expressed on the base of the difference in crosslinker and the experiment conditions (Burcea-Dragomiroiu et al., 2015). Results also showed that all the formulations have a narrow size distribution range with PI ranged between 0.0353 and 0.0581. Therefore, the results show that the βcyclodextrin based nanosponges have demonstrated a homogeneous size distribution. The zeta potential gives an idea about the type of charge on the surface of the prepared nanosponge formulations and so the stability of the prepared colloidal suspension. The zeta potential values are shown in Table 1. The zeta potential of all PXM-NS formulations was adequately high carrying a negative charge on their surfaces to produce stable colloidal suspension with minimal agglomeration (Gangadharappa et al., 2017).

### 4.2. The percentage encapsulation efficiency of PXM in nanosponges

The percentage encapsulation efficiency ranged between 35.33 ± 3.12 and 79.13 ± 4.33 for PXM-NS1 and PXM-NS10 respectively. The significant difference in PXM loading could be interpreted based on the degree of cross-linking which alters the incorporation ability of PXM. The Lowest drug loading was observed for formulation with low cross linker ratio (PXM-NS1, PXM-NS2, and PXM-NS3) while the highest drug loading was observed for crosslinker ratio one to eight. The higher the amount of crosslinker, the stronger the internal matrix structure with higher ability to entrap PXM, related results were reported for tamoxifen by S. Torne (Torne et al., 2013). Results also revealed that at the same crosslinker level a non-significant change in drug loading was observed with increasing the drug ratios. Shringirishi M. et al. also reported that the encapsulation of nifedipine in nanosponge did not affect by drug ratio (Huang et al., 2013).

### 4.3. Saturation solubility study for prepared PXM-NSs formulations

**Figure 2.**
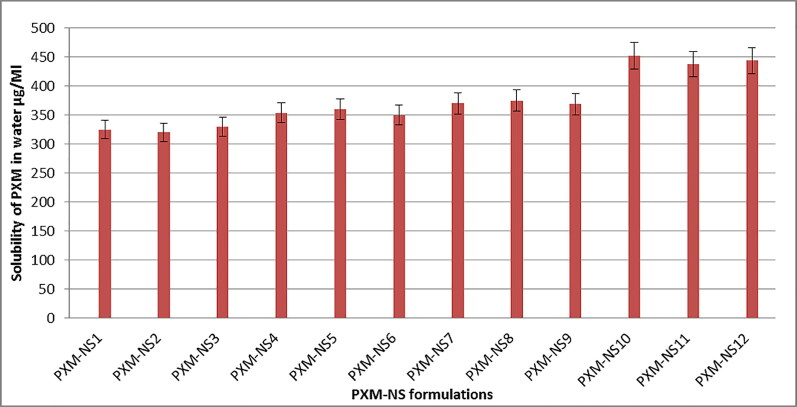
Saturation solubility of PXM-NS formulations in water.

The results showed that PXM nanosponges reported a significant increase in the solubility of PXM compared to the pure drug in distilled water. The solubility of the PXM was increased up to 23 folds in the prepared nanosponge formulations compared with the pure drug. The solubility of PXM in water was 19.4 ± 0.38 μg/mL, while its solubility in nanosponge particles was in the range of 320.19 ± 0.38 to 452.80 ± 0.92 µg/ml for PXM-NS2 and PXM-NS10 respectively which are represented in Figure 2. The results approve the ability of β-CD nanosponges to increase the solubility of PXM by forming a polymer mesh or network in the presence of crosslinker with nano size channels able to trap the drug molecules and increase its solubility. This structure favors drug molecule complexation and might help in increasing the drug solubility. The increase in the crosslinker concentration leads to a denser nanosponge matrix with higher ability to enhance the solubility of insoluble drug (Shringirishi et al., 2017).

### 4.4. Morphology study of PXM nanosponges using SEM

**Figure 3.**
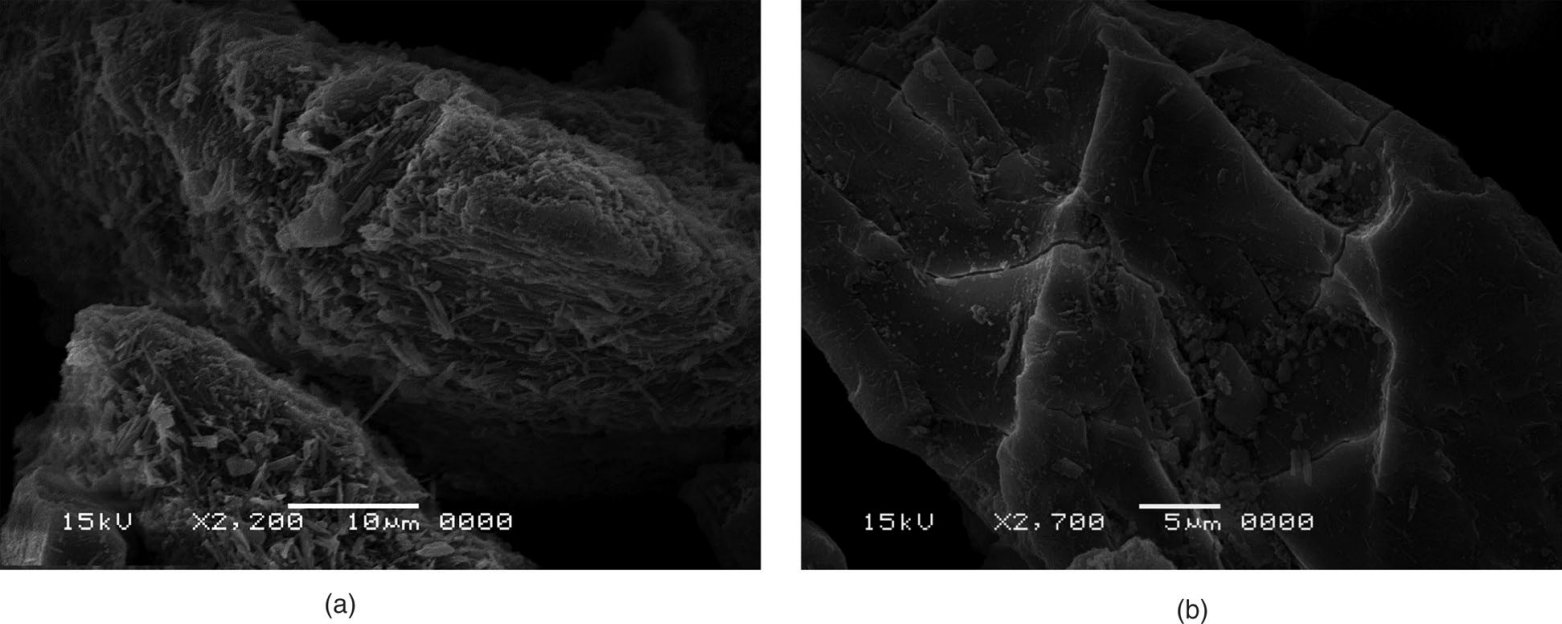
SEM image of PXM (a), and PXM-NS10 (b).

Figure 3 shows the SEM images of PXM crystals and drug-loaded nanosponges. PXM showed a sharp needle shape crystals with a smooth surface (Figure 3a). While drug loaded nanosponge showed a highly porous irregular surface exposing numerous spongy pores (Figure 3b).

### 4.5. FTIR study

**Figure 4.**
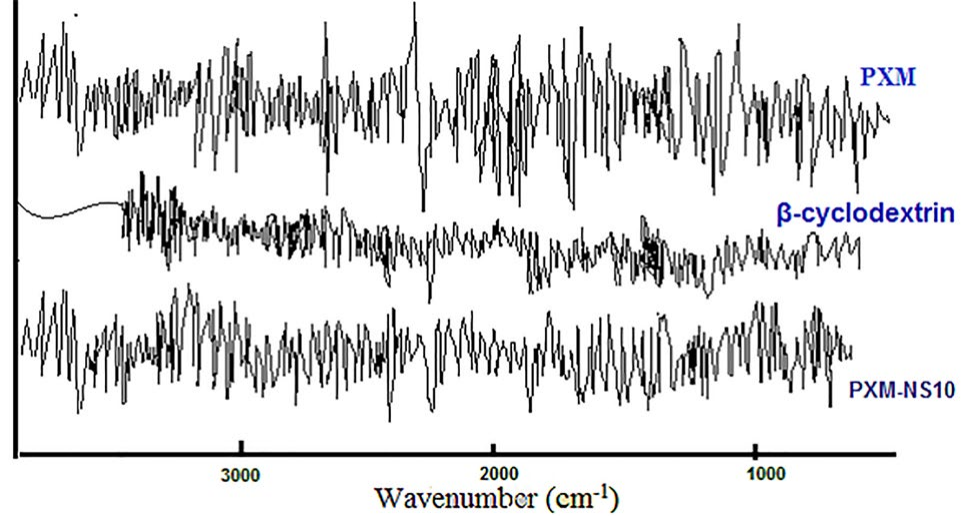
FTIR spectrum of PXM, β-cyclodextrin, PXM-NS10.

The FTIR spectrum of pure drug, β-cyclodextrin, and nanosponge formula is shown in Figure 4. The FTIR spectrum of PXM showed stretching band of N–H and O-H at 3390 cm^−1^ and 3250 cm^−1^ respectively. The spectrum showed a band at 773 cm^−1^ that corresponds to an aromatic ring. The pyridine group is represented by a peak at 1280 cm^−1^. β-cyclodextrin showed a characteristic peak at 3250–3390 cm^−1^ due to O-H stretching, an intense peak for C-H stretching at 2830 cm^−1^ also seen, in addition to a peak at 1650 cm^−1^ representing H-O-H group. In PXM nanosponges FTIR spectroscopy shows all stretching vibrations characteristic of PXM and β-cyclodextrin. So we can conclude that there was no chemical interaction in the nanosponge.

### 4.6. Differential scanning calorimetry study

**Figure 5.**
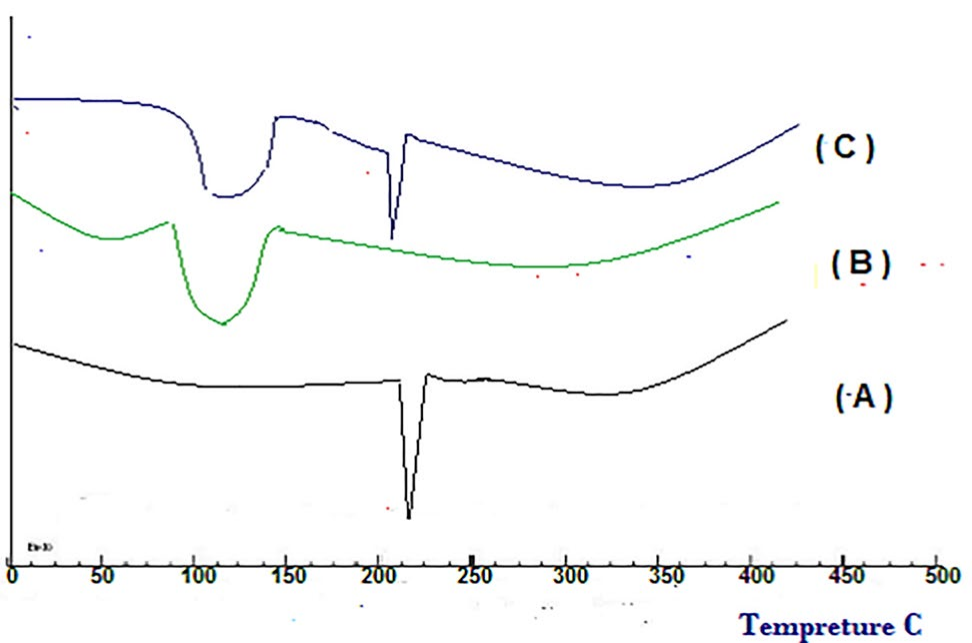
DSC thermograms of PXM (A), β-cyclodextrin (B), mixture 1:1 w/w of the drug and β-cyclodextrin (C).

DSC analysis is applied to determine any possible physical interaction between PXM and carrier. Piroxicam showed a sharp endothermic peak at 205.15 corresponding to its melting point (Figure 5A). β-cyclodextrin showed broad endothermic peak at 110.24 around its melting point (Figure 5B). Thermal behavior of the binary mixture 1:1 w/w of the drug and β-cyclodextrin showed a slight shift in PXM peak at 201.15 (Figure 5C), hence no interaction was found between the drug and the carrier.

### 4.7. In vitro dissolution study

**Figure 6.**
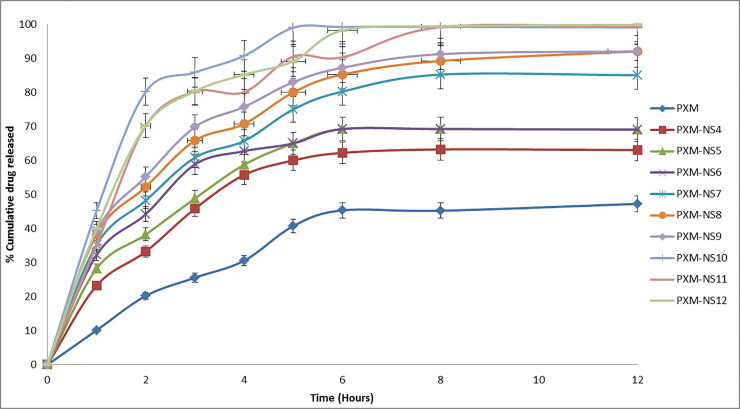
Cumulative % release of PXM and selected formulations of PXM-nanosponges.

Based on the encapsulation efficiency test; formulations have encapsulation efficiency of more than 50% were selected for further in vitro release study. The test was conducted in a gastric simulated fluid (pH 1.2) for two hours followed by ten hours in alkaline media (pH 7.4). The data were presented as percentage of cumulative drug released versus time. Dissolution profiles of pure PXM, and formulations (PXM-NS4–PXM-NS12) are shown in Figure 6. A significant enhancement in the dissolution rate of PXM from nanosponges in comparison to the pure drug was observed. All tested formulations showed a burst release of the drug within 30 minutes ranged between 20.23%±2.3 and 30.45%±3.5 for PXM-NS4 and PXM-NS10 respectively compared with 5.23%±3.0 for the pure drug. A significant difference between formulations was observed for the percentage of drug release after two hours PXM-NSs composed of one to six ratio of crosslinker showed 45.33 ± 2.6%, 48.41 ± 1.44% and 52.50 ± 1.5% of PXM release from PXM-NS4, PXM-NS5, and PXM-N6, respectively with about 69.31 ± 2.50% of the drug released after 12 hours. A significant increase at p level 0.5 was reported in the amount of drug released from formulations PXM-NS7, 8, and 9, and the results showed drug release ranged between 48.23 ± 3.0% and 55.67 ± 2.66% for PXM-NS7 and PXM-NS9 after two hours. The amount of drug released was about 86.15 ± 3.0%, 95.1 ± 2.39% and 89.64 ± 2.2% from PXM-NS10, PXM-NS11, and PXM-NS12, respectively within two hours. The results could be interpreted based on the amount of crosslinker used; the increasing its ratio relative to cyclodextrin leads to the formation of a porous fluffy structure with a higher ability to enhance dissolution and release of the drug. Many studies were reported the efficacy of cyclodextrin complex in the enhancement of the solubility of poorly soluble drugs using different crosslinkers and process conditions. H.V. Gangadharappa reported the enhancement of celecoxib release using β-cyclodextrin and NN-methylene bisacrylamide as a crosslinker and his experiment recorded a relationship between NN-methylene bisacrylamide concentration and the amount of drug released. Similar results were reported for taxifolin and paclitaxel by Shikov, A.N. et al., and Mognetti, B., et al., respectively (Mognetti et al., 2012; Shikov et al., 2009; European Federation of Pharmaceutical Industries Association and European Centre for the Validation of Alternative Methods, 2001).

### 4.8. Kinetic analysis of drug release

In order to define the release mechanism that gives the best description of the drug release pattern; the *in vitro* release data for all formulations (PXM-NS1–PXM-NS12) were fitted to kinetic equations models. The kinetic equations used were zero, first-order, Higuchi diffusion, and peppas model. Both the kinetic rate constant (k) and the determination coefficient (R^2^) were calculated and presented in Table 2.The best fit model with the highest determination coefficient (R^2^) value for all formulations were Higuchi diffusion model followed by Peppas model and finally zero-order. These results exposed that the kinetic pattern was best fitted to the Higuchi model which defines drug release from the polymeric box structure systems by diffusion mechanism. The Fickian diffusion (*n* < 0.5) mechanism of release was the release controlling mechanism for all formulations (Rao et al., 2013).

**Table 2.** The kinetic analysis study of PXM release data from nanosponges (PXM-NS1-12) according to four kinetic models.

Formula code	Zero order Q_t_ = Q_0_ + k_0_ t		First-order Q = Q_o_e^-kt^		Higuchi Q_t_ = k t^1/2^		Peppas Q_t_/Q_∞_ = kt^n^	
	R^2^	K (%min^-1^)	R^2^	K (min^-1^)	R^2^	K (%min^-1^)	R^2^	n
PXM-NS1	0.90	0.188	0.85	0.001	0.98	4.98	0.97	0.39
PXM-NS2	0.94	0.184	0.81	0.002	0.98	4.95	0.96	0.38
PXM-NS3	0.95	0.181	0.82	0.002	0.99	4.78	0.98	0.41
PXM-NS4	0.91	0.176	0.84	0.001	0.97	4.86	0.96	0.43
PXM-NS5	0.94	0.175	0.80	0.002	0.99	4.89	0.98	0.43
PXM-NS6	0.93	0.177	0.75	0.002	0.96	4.79	0.96	0.46
PXM-NS7	0.94	0.170	0.74	0.001	0.99	4.69	0.98	0.51
PXM-NS8	0.96	0.154	0.84	0.001	0.98	4.93	0.98	0.39
PXM-NS9	0.96	0.165	0.81	0.002	0.99	4.62	0.99	0.43
PXM-NS10	0.93	0.174	0.75	0.002	0.97	4.91	0.99	0.40
PXM-NS11	0.89	0.148	0.75	0.001	0.97	4.78	0.96	0.42
PXM-NS12	0.92	0.158	0.76	0.001	0.98	4.77	0.97	0.41

### 4.9. Stability study

**Figure 7.**
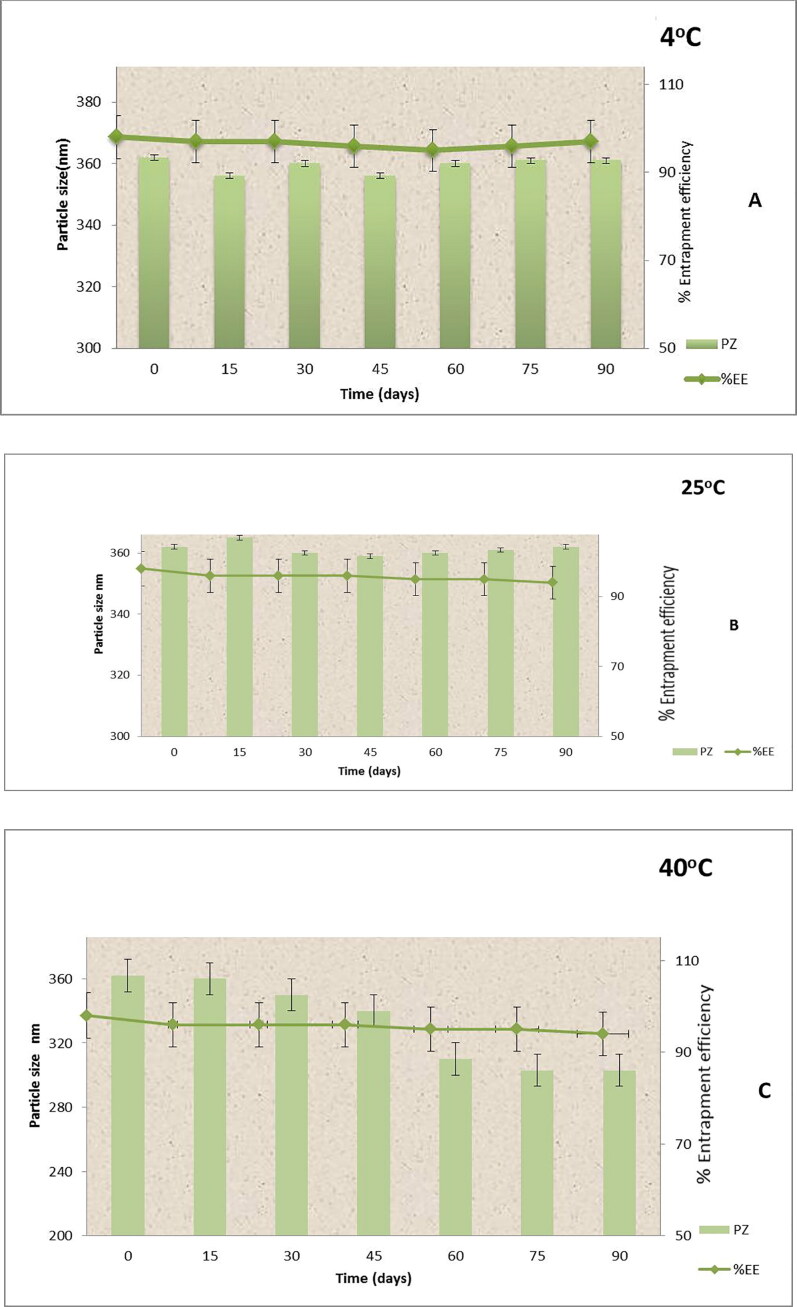
Stability results of PXM-NS10 in terms of both particle size and drug loading at (A) 4 °C, (B) 25 °C, and (C) 40 °C. Abbreviations: PZ, particle size; %EE, percentage entrapment efficiency.

The stability of nanosponges at three different storage conditions is an important point, to assess the influence of temperature on the probability of drug degradation and/or particle growth during storage. PXM-NS10 (selected as the optimal formula) was prepared at optimized conditions and studied for its stability at three temperatures namely 4 °C, 25 °C, and 40 °C. The selected formula was examined for its physical appearance, drug content, and particle size at intervals 0, 15, 30, 45, 60, 75, and 90 days. No obvious physical changes in either the color, the texture, or the appearance of the formula were observed during the test duration. Figure 7 displays the results of formula stability, it shows that there was no significant growth in the size or degradation was observed at 4 °C and 25 °C over 90 days (Figure 7A and 7B). Conversely, a decrease in the particle size with more than 50 nm, lowering in drug encapsulation, and darkening in particles color has occurred at 40 °C after 3 months as shown in Figure 7(C). The results could be understood on the base that; at high temperatures, particles lost part of their moisture content by evaporation which lead to over dryness of the particles and a decrease in its size in addition to the chemical degradation of PXM at this temperature (Higuchi, 1963).

### 4.10. Analgesic test using hot plate method

**Figure 8.**
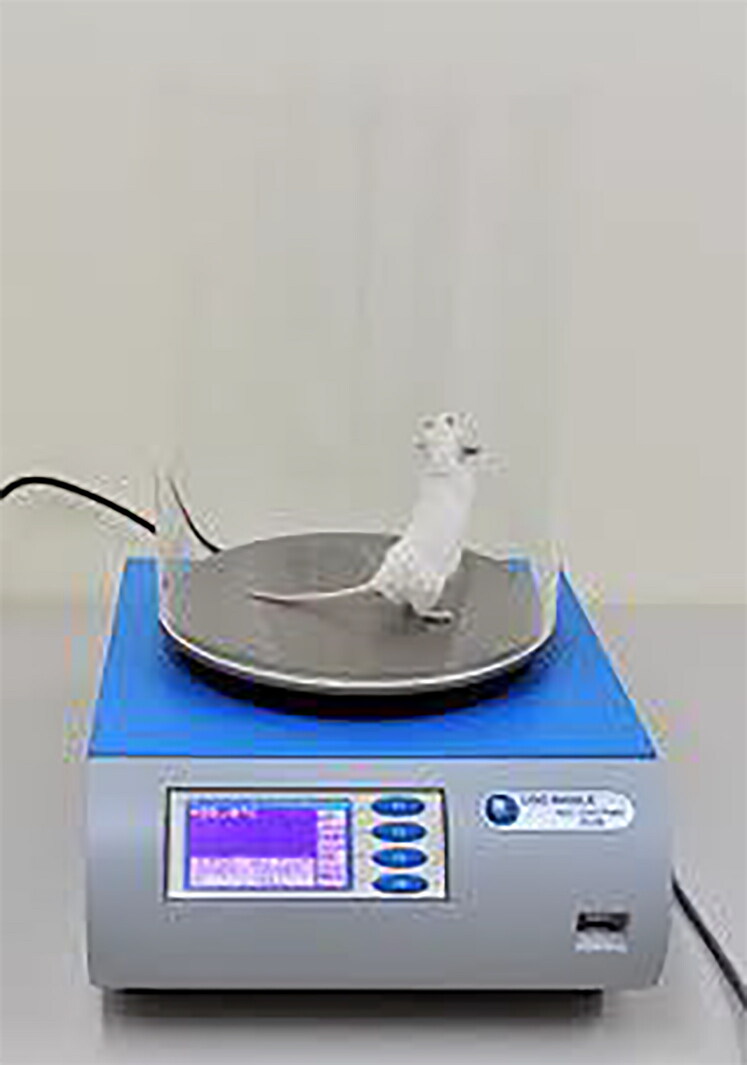
Hot plate analgesiameter apparatus.

**Figure 9.**
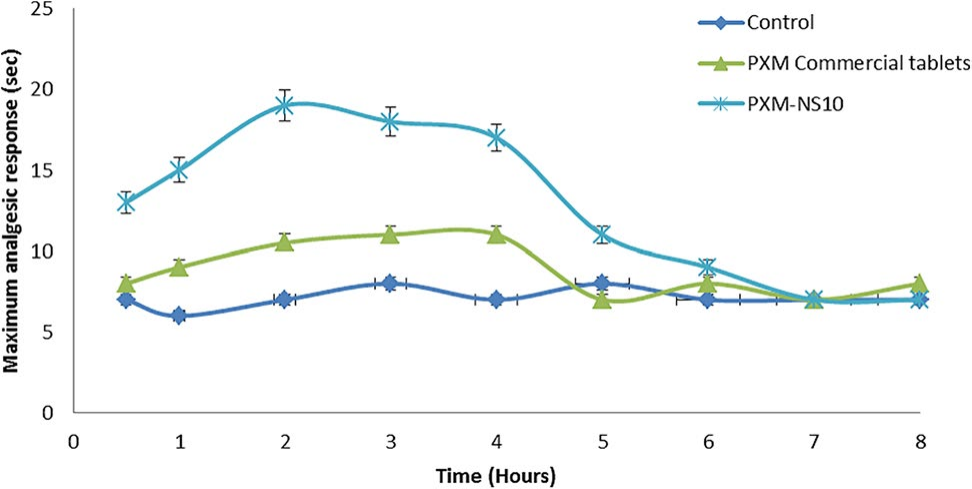
Analgesic activity of the selected formula of PXM nanosponges (PXM-NS10) compared with commercial tablets using the hot plate technique. All values are means ± SE.

PXM nanosponge formula (PXM-NS10) was selected to assess the analgesic activity of PXM in the comparison with PXM market tablets. The hot plate method was defined by Adams et al. science 1969 (Figure 8) (ICH Harmonized Tripartite Guidelines, 2003). This test was established for evaluating both the centrally and peripherally acting analgesics. Group III mice received normal saline (0.5 ml) and were used as a control for the assessment of the analgesic effect of PXM in the other groups. Three parameters have been used to evaluate the analgesic effect of PXM. These parameters are; MR (the maximum response) to PXM in terms of the time in seconds which reveals the intensity of response to the drug, TMR (the time of the maximum response) and DA (the duration of drug action) which represents the time which the effect is preserved. Figure 9 and Table 3 explained the effect of oral administration of PXM-NS10 and commercial PXM tablets on the variation of animal behavior toward pain stimulant (hot plate surface). The experiment showed that PXM commercial tablet showed a significant increase in analgesic activity (*p* < .05) in mice after 30 minutes of oral administration as compared to the control group and the MR was 10.3 ± 0.35 s was detected after 2 hours, and then the response decreased gradually till disappearance after 5 hours. On the other hand, oral administration of the selected formula (PXM-NS10) showed a significant increase in the analgesic responses (*p* < .05) in mice after 30 minutes of oral use as compared to the control group, and the maximum analgesic response, MR was 18.8 ± 0.75 s was reported after two hours then it decreased slowly till disappear after about 6 hours. This higher response and longer action may be due to the formulation characteristics which increase the solubility and absorption of PXM compared with traditional tablet dosage form (Adams et al., 1969).

**Table 3.** Parameters measured for the analgesic activity of PXM-NS10 and PXM commercial tablets in mice using hot plate test.

Groups	Parameters of analgesic activity		
	MR (sec) ± SE	TMR (hr)	DA (hr)
Control	7.2 ± 0.13	–	–
Commercial tablets	10.3 ± 0.35	2	4
PXM–NS10	18.8 ± 0.75	2	6

### 4.11. The in vivo bioavailability study

**Figure 10.**
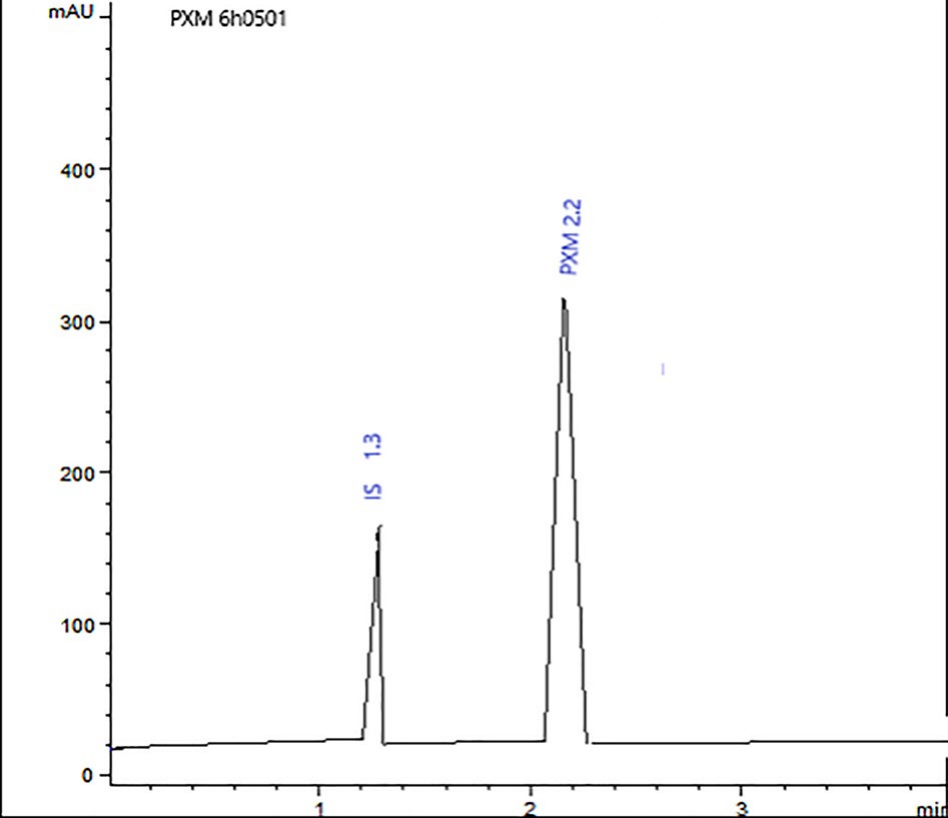
HPLC Spectrum of PXM and IS in plasma.

**Figure 11.**
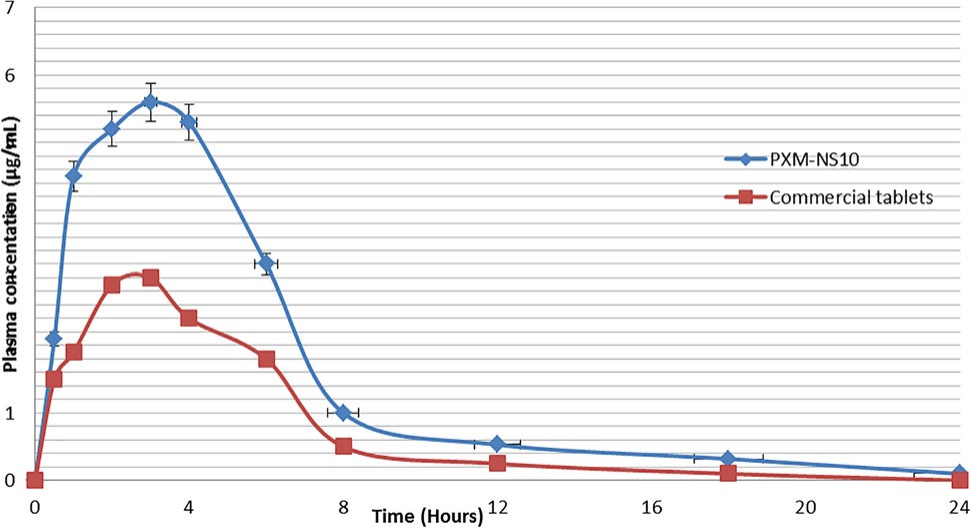
PXM plasma concentration time profiles in rats after oral administrations of PXM-NS10 and commercial tablets.

PXM-NS10 showed good results in particle size analysis, solubility test, in vitro release study, and stability results for that it was selected to be studied for in vivo performance. HPLC method was used to detect the amount of PXM in plasma (Figure 10) Plasma concentrations of PXM after oral administration of NS10 and commercial tablets showed that nanosponge formulation improved the absorption of PXM compared with commercial tablets. Maximum plasma concentrations of PXM were 5.52 ± 0.28 and 3.06 ± 0.66 μg/mL for NS10 and commercial tablets, respectively (Table 4 and Figure 11). There was about 1.8 fold increase in C_max_ of PXM from nanosponge as compared to commercial tablets. AUC—the area under the plasma concentration time curve—of PXM after oral administration of tablets was 19.82 ± 2.51 μg/mL h while the AUC of PXM after NS10 oral administration was 28.32 ± 1.6 μg/mL h. The time to achieve the maximum plasma concentration (t_max_) of PXM from both nanosponges and commercial tablets was not different at a significance level *p* < .5 (2.5 ± 0.10 h). The enhancement of PXM bioavailability after oral administration of NS could be explained based on the faster and higher absorption of PXM from NS compared with traditional tablets. This could be explained based on the enhancement in its solubility achieved by the small particle size, the large surface area, and the sponge structure formed by β-cyclodexterin (Cavalli et al., 2006; Ming-Wei et al., 2013). Nanostructures of Aceclofenac, Acyclovir and Nifidipine showed higher solubility, dissolution rate and finally better rate of absorption compared with traditional dosage forms (Dubes et al., 2003; Gangadharappa et al., 2017; Haroon et al., 2017).

**Table 4.** *In vivo* Pharmacokinetic Parameters of PXM after oral use of PXM-NS10 and commercial tablets in Rats.

Pharmacokinetic parameter	PXM-NS10	Commercial tablets
C_max_, µg/ml	5.52 ± 0.28	3.06 ± 0.66
T_max_, hr	2.5 ± 0.10	2.6 ± 0.09
AUC0-24 µgml^-1 ^hr	28.32 ± 1.6	19.82 ± 2.51
MRT, hr	10.6	9.8
Rel F (%)	142.88	–

## 5. Conclusion

Improving the analgesic effect of PXM was achieved by inclusion of the drug in β-cyclodextrin nanosponge. Twelve PXM-nanosponge formulations were designed using three ratios of β-cyclodextrin to cross linker and three ratios of drug to nanoponge to achieve the optimal nanosponge formula of PXM. The optimal condition was found at β-cyclodextrin to crosslinker ratio 1:8 and drug to nanoponge ratio 1:2. The in vitro solubility and dissolution study of PXM was improved noticeably by applying this formulation conditions. The optimum nanosponge formula of PXM showed a marked improvement in the analgesic activity and *in vivo* pharmacokinetic bioavailability compared to market tablets. The study showed that nanosponge formulated under optimal conditions can improve the analgesic activity of Piroxicam.
